# Association of enlarged perivascular spaces with upper extremities and gait impairment: An observational, prospective cohort study

**DOI:** 10.3389/fneur.2022.993979

**Published:** 2022-10-26

**Authors:** Yutong Hou, Shuna Yang, Yue Li, Wei Qin, Lei Yang, Wenli Hu

**Affiliations:** Department of Neurology, Beijing Chao-Yang Hospital, Capital Medical University, Beijing, China

**Keywords:** cerebral small vessel disease, enlarged perivascular space, gait, upper extremities, motor disturbance

## Abstract

**Background and objective:**

Gait disturbances are common in the elderly and can lead to the loss of functional independence and even death. Enlarged perivascular space (EPVS) and motor performance may be related, but only few studies have explored this relationship. The aim of our study was to investigate the effects of both the severity and location of EPVS on movement disorders.

**Method:**

Two hundred and six participants aged between 45 and 85 years old with complete magnetic resonance imaging (MRI) data were included in our analysis. EPVS were divided into basal ganglia (BG) and centrum semiovale (CSO), and their grades were measured. Gait was assessed quantitatively using a 4-m walkway and TUG test as well as semi-quantitatively using the Tinetti and SPPB tests. The function of upper extremities was evaluated by 10-repeat pronation–supination, 10-repeat finger-tapping, and 10-repeat opening and closing of the hands.

**Results:**

Both high-grade EPVS, whether in BG and CSO, were independently correlated with gait parameters, the TUG time, Tinetti, and SPPB tests. The EPVS located in BG had a significant association with 10-repeat finger-tapping time (β = 0.231, *P* = 0.025) and a similar association was also observed between CSO-EPVS and 10-repeat pronation–supination time (β = 0.228, *P* = 0.014).

**Conclusion:**

Our results indicated that EPVS was associated with gait disturbances, and a further investigation found that EPVS has an association with upper extremities disorder. EPVS should be considered as a potential target for delaying gait and upper extremities damage since CSVD can be prevented to some extent.

## Introduction

Gait impairments are prevalent among older adults and have important implications as they can increase the risks of falls, hospitalizations, and mortality (1). Among individuals 60 and older, these impairments are increasing at an alarming rate (2). Besides growing older, vascular risk factors also play an important role in gait disturbances (3). Cerebral small vessel disease (CSVD) characterized by white matter hyperintensity (WMH), lacunar, microbleeds, brain atrophy, and enlarged perivascular space (EPVS) has been widely considered to be the most important vascular causes of gait dysfunctions (4–8). There is extensive evidence confirmed that lacunar infarcts, WMH, and brain atrophy are associated with gait parameters disorders (5, 6, 9, 10). However, the motor impairment that is associated with CSVD cannot be fully explained by EPVS and need investigation.

The perivascular spaces (PVSs) are fluid-filled cavities surrounding the small penetrating cerebral arterioles and venules that drain waste products from the brain. They are considered dilated and functionally impaired when visible on MRI. Recent studies have shown that EPVS, especially those located in the basal ganglia (BG), may be related to cognition impairments and just a few studies have attempted to explore the relationship between EPVS and movement disorders. Some studies found that EPVS was associated with impaired motor function in patients with PD (11–13). But other studies suggested that EPVS has no impact on gait disorders (14–17). And all the studies assessed motor function using semi-quantitative scales that may not reflect changes in movement disorders accurately enough. Except for that, EPVS located in BG and centrum semiovale (CSO) may reveal different pathogenesis for gait disturbance. Thus, it is meaningful to investigate the relationship between EPVS in different regions and movement disorders and to intervene in risk factors.

Except for gait, hand function plays a crucial role in human activities and independent living, as well as influencing performance on tasks (18). Because upper extremity dysfunction does not present obvious symptoms and measurement is difficult, it is often underestimated. Only two studies have explored the influence of CSVD on upper extremities and found that WMH was associated with upper extremity disorder, but no association between EPVS and CSVD has been reported (19, 20).

Our study aims to examine the correlation between EPVS and gait and upper extremity function in BG and CSO independently in order to explore the pathogenesis of CSVD in movement disorders.

## Method

### Population

Two hundred and six consecutive participants for physical examination at the department of Neurology in Beijing Chao-Yang Hospital, Capital Medical University from February 2019 to July 2021 were enrolled in our study. Participants between 45 and 85 years of age could complete the movement tests and have their magnetic resonance imaging (MRI) data reviewed.

Participants were excluded from the study if they had a history of stroke or cancer, severe unrelated neurological diseases (including dementia, Parkinson's disease (PD) or PD-plus syndrome, aphasia, dysarthria, epilepsy); an intracranial space-occupying lesion; recent or current use of acetylcholine-esterase inhibitors, neuroleptic agents or L-dopa; severe arthritis or psychogenic gait disturbance, or inability finish the tests because of prominent visual, hearing, language impairment, or psychiatric disease.

In accordance with the Declaration of Helsinki, and participants signed informed consent for the use of their data for research, the Beijing Chao-Yang Hospital Institutional Review Board approved the study.

### Clinical characteristics collection

A wide range of demographic information (age, gender, height, weight), medical histories (hypertension, hypercholesterolemia, diabetes mellitus, ischemic stroke or transient ischemic attack, coronary heart disease, medication use), smoking, and alcohol history were collected. Hypertension was defined as blood pressure ≥140/90 mmHg or having a documented medical history and having been treated with medication; diabetes was defined as patients who had been informed of this diagnosis by a physician before admission or were receiving hypoglycemic treatments or hemoglobin A1C (HbA1C)≥6.5%; hyperlipidemia was defined as total cholesterol >5.2 mmol/L, TG ≥1.7 mmol/L, LDL >2.58 mmol/L or having a history of hyperlipidemia; previous ischemic stroke/TIA was defined as a history of related syndromes or documented information in the medical record. Laboratory parameters were collected on an empty stomach the following morning. We collected data on triglycerides, cholesterol, low-density lipoproteins (LDL), high-density lipoproteins (HDL), homocysteine, fasting blood glucose levels, glycosylated hemoglobin levels, etc.

### MRI data acquisition and evaluation

We acquired MRI data using 3.0-T MRI at Beijing Chao-Yang Hospital including the following sequence: T1-weighted imaging, T2-weighted imaging, fluid-attenuated inversion recovery imaging (FLAIR), diffusion-weighted imaging (DWI), and susceptibility-weighted imaging (SWI).

The EPVS was measured using T2-weighted images in BG and CSO. EPVS is a lesion that measures < 3 mm in diameter, which has the appearance of cerebrospinal fluid (CSF)-like signal (hyperintense on T2-weighted imaging, hypointense on FLAIR imaging) located surrounding the perforating arteries of the brain.

They might appear as round, oval, or linear-shaped lesions depending on the brain area and the plane sequence assessed (4). EPVS were counted in the slice and the side with the highest number. A 5-point visual rating ordinal scale (0, no EPVS; 1, 1–10 EPVS; 2, 11–20 EPVS; 3, 21–40 EPVS; 4, >40 EPVS) was used to evaluate the severity of EPVS in the CSO and BG (21). The severity of EPVS was dichotomized into low-grade (EPVS scores 0–1) and high-grade (EPVS scores 2–4) in our study.

White matter hyperintensity, lacunes, and microbleeds (CMBs) were diagnosed based on the Standards for Reporting Vascular Changes on Neuroimaging (STRIVE) criteria proposed in 2013 (4). Deep WMH (DWMH) and periventricular WMH (PVWMH) were graded on the Fazekas scale (each 0–3) using FLAIR and T2-weighted sequences (22). CMBs were defined as small (< 10 mm), homogeneous, and round foci of low signal intensity on SWI images (23). Lacunes were defined as small (< 20 mm in diameter), subcortical lesions of similar signal to CSF. Brain atrophy was assessed by the MRI visual ratings (24). The presence of CMB, lacunes, and brain atrophy was recorded.

Two investigators (Y-L, SN-Y) blinded to the clinical data independently reviewed the MRI and assessed the severity of EPVS, WMH, CMB, lacunar, and brain atrophy. An interobserver reliability test was performed on 50 subjects with a month's interval between the first and second readings. Kappa values for the intra-rater agreements were 0.81 to 0.86. The disagreement was resolved by the third investigator (W-Q, neuroradiologist).

### Assessment of motor performance

The gait assessment was conducted on a 4-m walkway. Participants were instructed to walk over the walkway at their own pace. A steady-state walking was ensured by starting 2 m in front of the walkway and stopping two meters behind it. Gait parameters were averaged over two walks: velocity (m/s), step length (cm) (distance between heel points in two consecutive footprints), number of steps (number of steps in 4-m walkway), and stride width (cm). Besides that, we also evaluated the TUG test time. Semi-quantitative assessments included the Tinetti test with 17 items (nine for balance and eight for gait) with a maximum score of 28 and the Short Physical Performance Battery (SPPB) assessing muscle strength, walking speed, and balance.

As for upper extremity function, 10-repeat pronation–supination test, 10-repeat finger-tapping test, and 10-repeat opening and closing of hands were used.

### Statistical analysis

Statistical analysis was performed using the SPSS 24.0 statistical package (SPSS Inc., Chicago, IL, USA) and Prism 9. Categorical variables as numbers and percentages, and continuous variables are presented as means and standard deviations, non-normal distributed variables as medians and interquartile ranges. Analyses of differences in motor performance and clinical information based on different burdens of EPVS in CSO and BG. One-way analysis of variance and Kruskal–Wallis test was used for continuous data, and chi-square test or Fisher exact test for categorical data.

The relationships between BG-EPVS and CSO-EPVS and motor performance were evaluated using binary logistic regression analysis with EPVS as a determinant and motor parameters (gait velocity, stride width, number of steps, step length, time of TUG, SPPB and Tinetti score, time of pronation–supination, time of finger-tapping and time of opening and closing hands) as outcome variables. Model 1 was adjusted for age, sex, height, and BMI, and Model 2 was the same as Model 1, with further adjustments for WMH judged by Fazekas score, presence of lacunar, brain atrophy, and CMB and other relevant risk factors. Statistical significance was established at *P* < 0.05.

## Result

### Demographic, imaging, and motor characteristics

The demographics, vascular risk factor variables, laboratory data, imaging markers of EPVS burden, and motor performance of the participants are shown in Table 1, Supplementary Table 1, and Table 2. A total of 206 patients were included in this study with a mean age of 60.3 ± 10.7 years, and 144.0 (69.9%) of the patients were male subjects. Among all the subjects included, 61 (29.2%) had high-grade BG-EPVS, 86 (41.4%) had high-grade CSO-EPVS, and 32 (15.5%) had both.

**Table 1:** Demographic, clinical, and CSVD characteristics of participants with different severities of EPVS.

**Variables**	**All (*n* = 206)**	**BG-EPVS**			**CSO-EPVS**		
		**Low-grade (*n* = 145)**	**High-grade (*n* = 61)**	***P***	**Low-grade (*n* = 120)**	**High-grade (*n* = 86)**	***P***
Age, year	60.3 ± 10.7	58.8 ± 10.2	63.8 ± 11.2	**< 0.001**	59.3 ± 12.0	61.7 ± 8.5	0.249
Sex, male, n (%)	144.0 (69.9)	100 (69.0)	44 (72.1)	0.651	85 (70.8)	59 (68.6)	0.731
Height, m	167.2 ± 8.3	167.4 ± 8.4	166.7 ± 7.8	0.600	166.9 ± 8.3	167.6 ± 8.2	0.516
Weight, kg	72.2 ± 10.0	72.4 ± 10.3	71.6 ± 9.1	0.670	71.3 ± 10.0	73.3 ± 9.8	0.127
BMI, kg/m^2^	25.8 ± 3.9	25.7 ± 3.2	26.0 ± 5.3	0.662	25.6 ± 4.6	26.1 ± 2.8	0.105
Hypertension, *n* (%)	131.0 (63.6)	87.0 (60.0)	44 (72.1)	0.099	73 (60.8)	58 (67.4)	0.331
Diabetes, *n* (%)	80.0 (38.8)	55.0 (37.9)	25.0 (41.0)	0.682	46.0 (38.3)	34.0 (39.5)	0.861
Hyperlipidemia, *n* (%)	37.0 (18.0)	24.0 (16.6)	13.0 (21.3)	0.417	16.0 (13.3)	21.0 (24.4)	**0.041**
CAD, *n* (%)	35.0 (17.0)	21.0 (14.5)	14.0 (23.0)	0.140	18.0 (15.0)	17 (14.6)	0.369
Ischemic stroke or TIA, *n* (%)	25.0 (12.1)	16 (11.0)	9.0 (14.8)	0.455	16.0 (13.3)	9.0 (10.5)	0.534
Smoke, *n* (%)	107.0 (51.9)	77 (53.1)	30 (49.2)	0.607	68.0 (56.7)	39.0 (45.3)	0.109
Alcohol, *n* (%)	86.0 (41.7)	60 (41.4)	26.0 (42.6)	0.869	54.0 (45.0)	32.0 (37.2)	0.263
PVWMH score (0–3)	1.0 (0, 2.0)	1.0 (0,1.0)	1.0 (1.0,2.0)	**< 0.001**	1.0 (0,1.0)	1.0 (1.0,2.0)	**0.005**
DWMH score (0–3)	0 (0,1.5)	0 (0,1.0)	1.0 (0,2.0)	**< 0.001**	0 (0,1.0)	1.0 (0,1.5)	**0.006**
Fazekas score	1.0 (0,3.0)	1.0 (0,2.0)	3.0 (1.0,4.0)	**< 0.001**	1.0 (0,3.0)	1.0 (1.0,3.0)	**0.013**
Presence of CMBs, *n* (%)	78.0 (34.8)	46.0 (28.4)	32.0 (51.6)	**0.001**	40.0 (30.1)	38.0 (41.8)	**0.041**
Presence of lacunes, *n* (%)	96.0 (43.0)	57.0 (35.4)	39.0 (62.9)	**< 0.001**	41.0 (30.8)	55.0 (61.1)	**< 0.001**
Presence of brain atrophy, *n* (%)	116.0 (51.8)	69.0 (42.6)	47.0 (75.8)	**< 0.001**	55.0 (41.4)	61.0 (67.0)	**< 0.001**

Data represent the number (percentage), mean±standard deviation, or median (interquartile range).

CSVD, cerebral small vessel disease; EPVS, enlarged perivascular space; BG-EPVS, basal ganglia-EPVS; CSO-EPVS, centrum semiovale-EPVS; BMI, body mass index; CAD, coronary artery disease; TIA, transient ischemic attack; PVWMH, periventricular white matter hyperintensity; DWMH, deep white matter hyperintensity; CMB, cerebral microbleed.

Bold values indicate the significant difference.

**Table 2:** Movement data of participants with different severities of EPVS.

**Variables**	**All (*n* = 206)**	**BG-EPVS**			**CSO-EPVS**		
		**Low-grade (*n* = 145)**	**High-grade (*n* = 61)**	***P***	**Low-grade (*n* = 120)**	**High-grade (*n* = 86)**	***P***
Step length, cm	64.7 (58.0, 68.0)	66.7 (60.0, 69.3)	60.0 (54.6, 66.1)	0.001	66.7 (60.0, 70.3)	61.1 (55.0, 66.8)	0.001
Stride width, cm	10.8 (8.0, 14.0)	10.0 (7.0, 13.0)	13.0 (10.0, 15.0)	< 0.001	10.0 (7.0, 12.3)	13.0 (9.5, 15.0)	< 0.001
Number of steps, steps	6.3 (5.9, 7.0)	6.0 (5.8, 6.7)	6.7 (6.0, 7.8)	< 0.001	6.0 (5.8, 6.8)	6.6 (5.9, 7.3)	0.034
Gait velocity, m/sec	1.1 (1.0, 1.2)	1.1 (1.0, 1.2)	1.0 (0.9, 1.1)	< 0.001	1.1 (1.0, 1.2)	1.0 (0.9, 1.2)	0.006
Tinetti test (range 0–28)	26.0 (24.2, 28.0)	28.0 (26.0, 28.0)	25.0 (24.3, 26.0)	< 0.001	28.0 (25.0, 28.0)	25.0 (23.0, 27.0)	< 0.001
Body balance (range 0–16)	15.0 (14.0, 16.0)	16.0 (14.0, 16.0)	14.0 (13.0, 14.0)	< 0.001	16.0 (14.0, 16.0)	14.0 (13.0, 15.0)	< 0.001
Gait (range 0–12)	12.0 (11.0, 12.0)	12.0 (11.0, 12.0)	11.0 (11.0, 11.0)	< 0.001	12.0 (11.0, 12.0)	11.0 (11.0, 12.0)	< 0.001
TUG test, sec	9.4 (8.3, 10.9)	8.7 (8.1, 10.1)	10.6 (9.6, 12.1)	< 0.001	8.9 (8.1, 10.2)	10.0 (8.6, 11.1)	0.003
SPPB test (range 0–12)	11.0 (9.0, 12.0)	12.0 (10, 12.0)	9.0 (8.0, 11.0)	< 0.001	12.0 (10.0, 12.0)	10.0 (9.0, 11.0)	< 0.001
Standing balance (range 0–4)	3.0 (2.0, 4.0)	4.0 (3.0, 4.0)	2.0 (2.0, 3.0)	< 0.001	4.0 (2.0, 4.0)	3.0 (2.0, 4.0)	< 0.001
Timed walk (range 0–4)	4.0 (4.0, 4.0)	4.0 (4.0, 4.0)	4.0 (4.0, 4.0)	0.036	4.0 (4.0, 4.0)	4.0 (4.0, 4.0)	0.226
Repeated chair stands (range 0–4)	4.0 (3.0, 4.0)	4.0 (3.5, 4.0)	3.0 (3.0, 4.0)	< 0.001	4.0 (3.0, 4.0)	3.0 (3.0, 4.0)	< 0.001
Mean pronation-supination, sec	6.7 (5.4, 7.7)	6.3 (5.0, 7.3)	7.3 (6.7, 8.1)	< 0.001	6.0 (4.8, 7.4)	7.2 (6.7, 7.9)	< 0.001
Left hand pronation-supination, sec	6.8 (5.3, 7.7)	6.3 (5.1, 7.4)	7.3 (6.8, 8.2)	< 0.001	6.0 (4.9, 7.5)	7.2 (6.8, 7.9)	< 0.001
Right hand pronation-supination, sec	6.6 (5.2, 7.7)	6.1 (4.9, 7.2)	7.3 (6.5, 8.1)	< 0.001	6.0 (4.8, 7.3)	7.2 (6.3, 7.9)	< 0.001
Mean finger-tapping, sec	4.6 (3.8, 6.1)	4.2 (3.7, 5.5)	5.7 (4.4, 6.8)	< 0.001	4.2 (3.5, 5.5)	5.4 (4.2, 6.3)	< 0.001
Left hand finger-tapping, sec	4.5 (3.8, 6.1)	4.3 (3.6, 5.6)	5.8 (4.2, 6.8)	< 0.001	4.1 (3.5, 5.7)	5.4 (4.2, 6.2)	< 0.001
Right hand finger-tapping, sec	4.7 (3.8, 6.1)	4.2 (3.7, 5.6)	5.6 (4.7, 7.0)	< 0.001	4.1 (3.5, 5.5)	5.4 (4.2, 6.2)	< 0.001
Mean opening and closing hands time, sec	4.6 (4.1, 5.8)	4.4 (4.1, 5.2)	5.4 (4.3, 6.7)	0.001	4.4 (3.9, 5.2)	5.0 (4.3, 6.2)	0.002
Left hand opening and closing hands time, sec	4.8 (4.1, 5.7)	4.5 (4.1, 5.2)	5.4 (4.3, 6.4)	0.001	4.5 (4.0, 5.3)	5.0 (4.3, 6.3)	0.001
Right hand opening and closing hands time, sec	4.6 (4.0, 5.7)	4.4 (4.0, 5.3)	5.4 (4.2, 6.7)	0.001	4.3 (4.0, 5.3)	5.0 (4.2, 6.3)	0.005

Data represent the number (percentage), mean ± standard deviation, or median (interquartile range).

TUG, Timed-Up-and-Go; SPPB, Short Physical Performance Battery.

The median walking speed was 1.1 m/s, the step length was 64.7 cm, the stride width was 10.8 cm, the number of steps was 6.3 steps in a 4-m walkway, and the median time for the TUG test was 9.4 s. The median scores for Tinetti and SPPB semi-quantitative scales are 26 and 11, respectively. The data for evaluating upper limb function were 6.7, 4.6, and 4.6 s for median 10-repeat pronation–supination, 10-repeat finger-tapping, and 10-repeat opening and closing hands time, respectively.

### Comparison of risk factors and motor function stratified by EPVS severity

In the BG-EPVS group, patients with high-grade BG-EPVS were older (63.8 ± 11.2 vs. 58.8 ± 10.2, *P* < 0.001) and had higher neutrophil counts (63.0 ± 13.5 vs. 59.1 ± 8.8, *P* = 0.021). In terms of CSVD, significant differences were found in WMH, CMB, lacunar, and brain atrophy. In addition, the high-grade BG-EPVS group showed significantly worse motor function in gait and upper extremities, and had lower Tinetti and SPPB scores, all *P* < 0.05.

In the CSO-EPVS group, the high-grade CSO-EPVS burden group had a higher proportion of hyperlipidemia (24.4 vs. 13.3%, *P* = 0.041) and higher burden of WMH, CMB, lacunar, and brain atrophy than low-grade CSO-EPVS burden group. There were still significant differences in gait and upper extremity function between high-grade CSO-EPVS and low-grade CSO-EPVS groups.

### Association between BG-EPVS and gait and upper extremities functions

**Figure 1:**
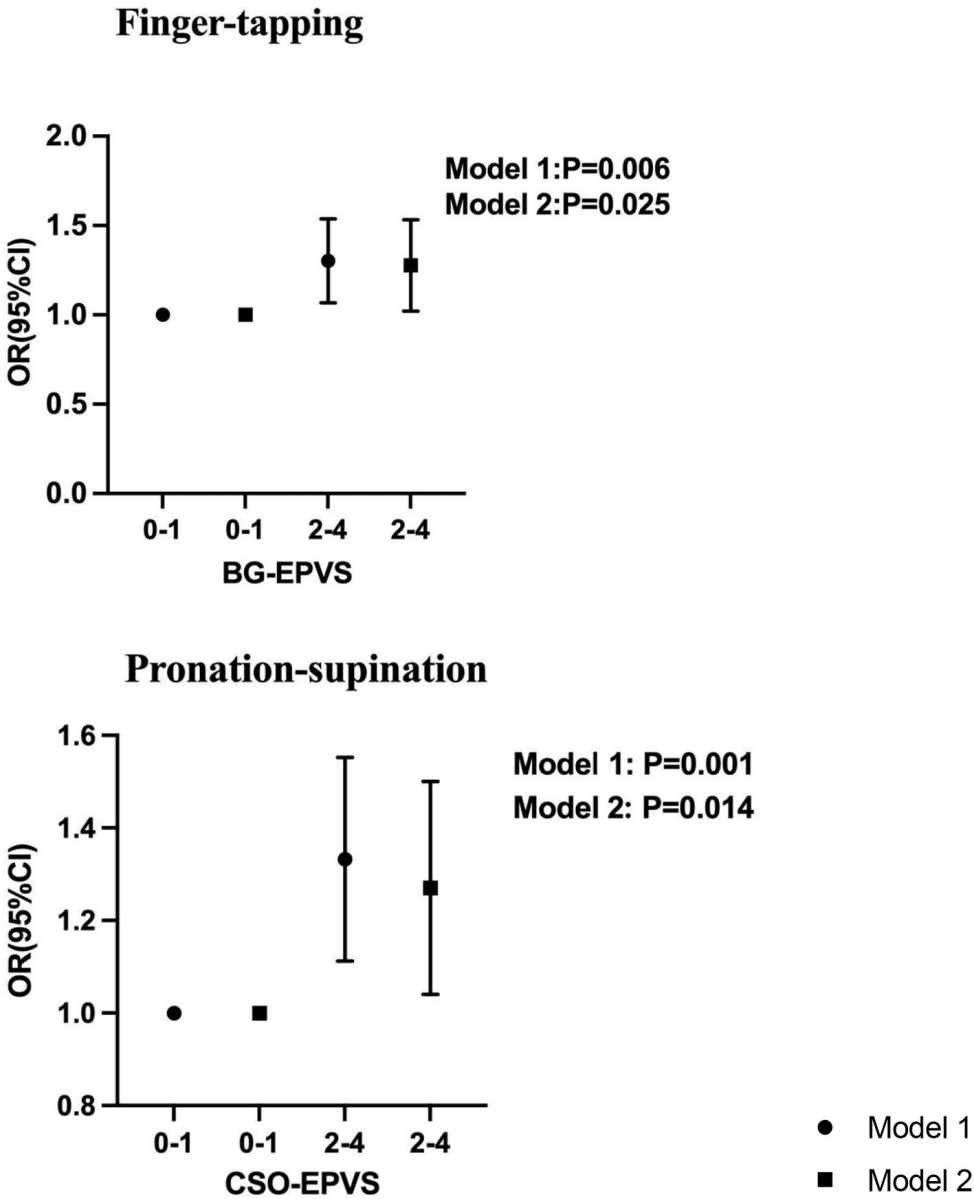
Association between BG-EPVS and CSO-EPVS and impaired upper extremity function by logistic regression. Model 1 was adjusted for age, sex, height, and BMI. Model 2 is with additional adjustment for hypertension, diabetes, hyperlipidemia, neutrophil, hemoglobin, Fazekas score, the present of lacunar, brain atrophy, and CMB.

High-grade BG-EPVS, adjusted for sex, age, height, and BMI, was associated with step length, velocity, number of steps, stride width, TUG time, and Tinetti and SPPB scores. The 10-repeat pronation–supination, 10-repeat finger-tapping, and 10-repeat opening and closing hands time were also found to be significantly correlated with high-grade BG-EPVS, as shown in Table 3 and Figure 1. Model 2 showed significant associations between high-grade BG-EPVS and step length, stride velocity, TUG time, and 10-repeat finger-tapping time, after additional adjustments for WMH, presence of lacunar, brain atrophy and CMB and relevant vascular risk factors (β = −0.037, *P* = 0.041; β = −3.606; *P* = 0.003; β = 0.185; *P* = 0.003; β = 0.231; *P* = 0.025). And correlations between semi-quantitative gait and balance tests, including Tinetti and SPPB, and severe BG-EPVS were found (β = −0.562, *P* = 0.001; β = −0.492; *P* < 0.001). But there were no associations found in stride width and any other assessments of upper limbs.

**Table 3:** Logistic regression analyses for the association between EPVS burden and movement disorder.

**Variables**	**BG-EPVS**				**CSO-EPVS**			
	**Model 1 β coefficient (95% CI)**	***P***	**Model 2 β coefficient (95% CI)**	***P***	**Model 1 β coefficient (95% CI)**	***P***	**Model 2 β coefficient (95% CI)**	***P***
Step length, cm	−0.056 (0.907, 0.986)	0.009	−0.037 (0.796, 0.998)	**0.041**	−0.074 (0.892, 0.966)	< 0.001	−0.068 (0.896, 0.974)	**0.001**
Stride width, cm	0.149 (1.067,1.263)	0.001	0.072 (0.976, 1.183)	0.142	0.181 (1.107, 1.298)	< 0.001	0.163 (1.074, 1.289)	**< 0.001**
Number of steps, steps	0.407 (1.126,2.003)	0.006	0.287 (0.999, 1.776)	**0.050**	0.170 (0.937,1.501)	0.157	0.134 (0.887, 1.476)	0.300
Gait velocity, m/sec	−4.671 (0.001, 0.088)	< 0.001	−3.606 (0.002, 0.298)	**0.003**	0.017 (0.552,1.874)	0.956	0.181 (0.641, 2.243)	0.571
Tinetti test	−0.589 (0.451, 0.856)	< 0.001	−0.562 (0.398, 0.869)	**0.001**	−0.510 (0.556, 0.855)	< 0.001	−0.499 (0.514, 0.863)	**0.001**
Body balance	−0.551 (0.458, 0.725)	< 0.001	−0.418 (0.499, 0.868)	**0.003**	−0.448 (0.520, 0.785)	< 0.001	−0.427 (0.502, 0.847)	**0.001**
Gait	−2.090 (0.058, 0.263)	< 0.001	−1.707 (0.079, 0.415)	**< 0.001**	−1.604 (0.110, 0.368)	< 0.001	−1.612 (0.096,0.413)	**< 0.001**
TUG test, sec	0.279 (1.136,1.538)	< 0.001	0.185 (1.018, 1.423)	**0.003**	0.139 (1.012,1.305)	0.032	0.097 (1.050, 1.279)	**0.034**
SPPB test	−0.550 (0.467, 0.712)	< 0.001	−0.492 (0.481, 0.776)	**< 0.001**	−0.364 (−0.579, −0.834)	< 0.001	−0.365 (0.557, 0.865)	**0.001**
Standing balance	−0.832 (0.310, 0.611)	< 0.001	−0.663 (0.355, 0.747)	**< 0.001**	−0.458 (0.474, 0.843)	0.002	−0.405 (0.476, 0.933)	**0.018**
Repeated chair stands	0.378 (1.234, 1.726)	< 0.001	0.338 (1.160, 1.694)	**< 0.001**	0.265 (1.128, 1.506)	< 0.001	0.258 (1.095, 1.530)	**0.002**
Mean pronation-supination, sec	0.287 (1.117, 1.588)	0.001	0.158 (0.974, 1.410)	0.094	0.278 (1.119, 1.558)	0.001	0.228 (1.048, 1.507)	**0.014**
Left hand pronation-supination, sec	0.309 (1.136, 1.634)	0.001	0.175 (0.984, 1.442)	0.072	0.313 (1.150, 1.627)	< 0.001	0.260 (1.071, 1.572)	**0.008**
Right hand pronation-supination, sec	0.250 (1.091, 1.510)	0.003	0.135 (0.961, 1.362)	0.130	0.233 (1.084, 1.470)	0.003	0.189 (1.023, 1.428)	**0.026**
Mean finger-tapping, sec	0.253 (1.074, 1.544)	0.006	0.231 (1.030, 1.541)	**0.025**	0.291 (1.099, 1.627)	0.004	0.154 (0.954, 1.428)	0.133
Left hand finger-tapping, sec	0.262 (1.084, 1.558)	0.005	0.247 (1.042, 1.574)	**0.019**	0.324 (1.136, 1.682)	0.001	0.194 (0.980, 1.504)	0.076
Right hand finger-tapping, sec	0.232 (1.055, 1.509)	0.011	0.201 (1.008, 1.484)	**0.041**	0.246 (1.055, 1.550)	0.012	0.112 (0.933, 1.340)	0.225
Mean opening and closing hands time, sec	0.266 (1.059, 1.606)	0.012	0.179 (0.968, 1.477)	0.097	0.187 (0.998, 1.457)	0.052	0.144 (0.949, 1.406)	0.150
Left hand opening and closing hands time, sec	0.310 (1.101, 1.690)	0.005	0.233 (1.009, 1.579)	0.052	0.230 (1.032, 1.536)	0.059	0.196 (0.985, 1.503)	0.069
Right hand Opening and closing hands time, sec	0.209 (1.015, 1.497)	0.035	0.124 (0.938, 1.365)	0.197	0.139 (0.968, 1.365)	0.113	0.095 (0.924, 1.308)	0.287

β, standardized β coefficient.

Model 1 is with adjustment for age, sex, height, and BMI.

Model 2 is with additional adjustment for hypertension, diabetes, hyperlipidemia, neutrophil, hemoglobin, Fazekas score, the present of lacunar, brain atrophy, and CMB.

Bold values indicate the significant difference.

### Associations between CSO-EPVS and gait and upper extremities functions

According to Model 1, high-grade CSO-EPVS correlated with wider stride widths, shorter step lengths, longer TUG time, and poorer performance on Tinetti, SPPB, and upper extremity tests, all *P* < 0.05. A significant association between high-grade CSO-EPVS and impaired gait function remained after adjusting for additional confounders in Model 2. The patients with severe CSO-EPVS were more likely to have impaired stride width and step length, and strong correlations were found (β = −0.163, *P* < 0.001; β = −0.068, *P* = 0.001). And the relationship between pronation–supination and severe CSO-EPVS was still obvious (β = 0.228, *P* = 0.014).

## Discussion

In a population-based study, we investigated the association between EPVS and gait and upper extremity functions. There was a correlation between high-grade BG-EPVS and shorter step length, lower stride velocity, and poor performance in the TUG, Tinetti, and SPPB tests. High-grade CSO-EPVS was associated with wider stride width, shorter step length, longer time for the TUG test, and a lower score on the Tinetti and SPPB tests. A novel finding in our study was the correlation between high-grade BG-EPVS and 10-repeat finger-tapping time and high-grade CSO-EPVS and 10-repeat pronation–supination. These results implied that EPVS may deteriorate gait and upper extremity function and should be observed and managed appropriately in CSVD treatment.

According to the clinical data, we found that there was a clear difference in neutrophils and hemoglobin between low-grade and high-grade BG-EPVS burdens. The increasing neutrophils might be a consequence of chronic systemic inflammation in CSVD, which activates interleukin production (25, 26) and systemic and vascular inflammatory status have also been found to be associated with CSVD development and prognosis (27). Hemoglobin has been suggested to not only reflect oxygen consumption but also be related to blood viscosity, which is associated with stroke risk when increased (28). BG-EPVS was associated with hypertensive arteriopathy, impaired normal perivascular cerebral blood flow, and damaged the blood–brain barrier (BBB). The dysfunction of BBB allows substances normally found in the blood to enter extravascular tissues. The high hemoglobin level, as well as the increased blood viscosity, further aggravates the damage to the BBB and the extravasation of blood components, ultimately leading to EPVS (29, 30). Therefore, increased hemoglobin could be a reasonable risk factor for EPVS.

Several previous studies have demonstrated that CSVD, including WMH, lacunes, and microbleeds, are associated with gait disorders, but the relationship between EPVS and motor function has rarely been explored. The study from Tian Tan Hospital examined the association between EPVS and motor dysfunction in patients with Parkinson's disease based on MDS-UPDRS III scores, finding that EPVS was not associated with rigidity, bradykinesia, or tremor (7, 16). While the study by Shin et al. examined the relationship between BG-EPVS, white matter EPVS, and motor function in patients with Parkinson's disease and concluded that severe BG-EPVS was associated with worse motor symptoms (12). Another study by Chung et al. found that EPVS participants exhibited a more severe decrease in dopamine transporters and a greater risk of freezing gait with PD (13). However, these studies have focused on patients with Parkinson's disease, and it is not known whether it has a similar effect in normal older adults.

Some research was done on normal elderly individuals except those with Parkinson's disease. In Shun Yi study, a community-based study explored the relationship between EPVS and gait dysfunction, but no positive correlation was found (19). The results of another study from Shanghai indicated that EPVS was significantly correlated with poorer performance in the Tinetti test after adjusting for age, sex, and height, but they were not sustained after further adjustments, and no association was found between EPVS and gait quantitative parameters (8). In our study, we assessed the function of gait with both quantitative and semi-quantitative assessments in normal elder people and found that stride width and step length were correlated with CSO-EPVS, step length, and stride velocity were correlated with BG-EPVS, and both BG-EPVS and CSO-EPVS performed poorly on Tinetti and SPPB tests. In analyzing the possible causes, we found that the incidence of EPVS in the Shanghai study was lower than in our study, which could affect the discovery of movement disorders. Another limitation of the study, the lesions of EPVS were not differentiated, which may reduce the specificity between EPVS and gait. The EPVS in BG and CSO and motor dysfunction has not been specifically studied, and that would be of interest to explore.

A distinct topographic pattern of EPVS is associated with different types of microangiopathy; CSO-EPVS is associated with CAA, whereas BG-EPVS is associated with vascular factors. According to previous studies across different populations, BG-EPVS severity is strongly associated with hypertension, WMH, and deep lacunes believed to be vascular in origin (31). The RUN DMC study concluded that WMH in the BG and lacunar infarcts in the thalamus are associated with lower velocities (32). And the latest study found that large numbers of BG-EPVS were related to lower gait speed and shorter stride length (33), which is consistent with our results. The degree of CSO-EPVS is associated with a higher incidence of CAA intracerebral hemorrhage, as well as lobar CMB (34). The relationship between cerebral Aβ deposition and reduced gait speed, muscle strength, and balance in older adults with cognitive impairment has been declared (35). At the same time, the presence of CMB was independently associated with shorter strides and worse Tinetti and TUG performance, particularly in temporal, frontal lobes, and thalamus (36). The association between CSO-EPVS and movement disorders is still unclear, so our study is valuable.

With aging, changes in motor function, especially upper limb function, affect independent living, quality of performance, and completion of daily activities (18). Several studies have established the relationship between CSVD and gait disturbances, but very few have examined the relationship between CSVD and upper extremities, particularly EPVS. Research from the University of British Columbia examined the function of upper extremity after chronic stroke using the Fugl-Meyer assessment and Wolf Motor Function Test (WMFT), and concluded that deep WMH volume best predicts upper limb impairment (37). Another study from Birmingham suggested that the WMH volume might be important in evaluating stroke-related upper extremity motor function based on the Motor Activity Log and WMFT (38). These studies had a small sample size, and used motor assessments for patients with post-stroke, which cannot eliminate all effects of stroke on upper limbs, and the applicability of the scale to patients with CSVD was unknown. Furthermore, it is unknown whether other categories of CSVD have relationships with upper limbs. The Shunyi study detected the relationship between upper extremities function and CSVD and had a conclusion that WMH was related to upper extremity disorder, but EPVS was not (19). Our study found that both BG-EPVS and CSO-EPVS were associated with upper extremity disorders using a measurement tool more appropriate for functional measures in patients with CSVD, following strict adjustment for WMH, lacunes, CMB, and brain atrophy, which could help explore the relationship between motor dysfunction and EPVS more accurate.

At present, there is no clear understanding of the mechanism of how EPVS leads to motor dysfunction. The ability to move, balance, and walk is dependent upon the integrity of a complex motor-circuit network that connects the brain stem and the BG to the frontal cortex (39). PVSs are associated with CSF circulation and perineural lymphatic drainage (40), are part of the blood–brain barrier (BBB), and contain scavenger cells that remove waste from the brain. These processes play a very important role in maintaining homeostasis, which ultimately contributes to the brain's immune surveillance. EPVSs are thought to be caused by perivascular blockages that disrupt PVS normal functions. EPVS lead to the accumulation of toxic metabolic products that damage the microenvironment of the brain, disrupt fibers, and disrupt brain networks, eventually causing movement dysfunction (41). Studies have found that the BG–thalamocortical circuit, brain-network efficiency, and loss of WM-microstructure integrity are involved in gait impairment in subjects with CSVD (9, 42). And global EPVS, especially BG-EPVS, have been found to have an association with increasing WMH burden (43). This show that the BG-EPVS have a broad effect on the whole brain and induce both lower and upper extremity motor dysfunction. However, the current research was not sufficient, and further investigation was considered.

In summary, we found that EPVS both in BG and CSO are related to gait and upper extremities disorder, and few studies were investigated and mostly negative. In addition, we found some interesting risk factors including neutrophils and hemoglobin had correlations with EPVS which needed more attention. Several limitations involved in this study should be considered. First, the cross-sectional design may be affected by recall bias, and the hospital-based study may affect the generalizability of the result. Future longitudinal studies will be needed. Second, the assessment of EPVS, WMH, CMB, lacunes, and brain atrophy were not accurately measured; better imaging equipment and software packages are required to assess CSVD quantitatively in order to explore the correlation more rigorously. Third, cognitive function and muscle strength were not assessed in our study, and the influence of cognitive function and muscle strength on the results should be further ruled out. Fourth, the sample size was relatively small, which limits the statistical power; thus, the findings of the present study should be considered preliminary, and multicenter prospective cohort study with a large sample should be performed.

## Conclusion

Our study confirmed that EPVS had an association with both gait and upper extremity function disorders. The presence of EPVS seems to be another driving force for gait impairment in healthy elderly subjects. These findings should be confirmed in a larger study.

## Data availability statement

The raw data supporting the conclusions of this article will be made available by the authors, without undue reservation.

## Ethics statement

The studies involving human participants were reviewed and approved by the Ethics Committee of Beijing Chao-Yang Hospital, Capital Medical University. The patients/participants provided their written informed consent to participate in this study.

## Author contributions

YH drafted the manuscript and conducted the statistical analyses. WH managed the database and provided additional statistical expertise. YH, SY, YL, WQ, and LY contributed to the conception, design of the study, and interpretation of the data. YL and SY provided expertise in brain imaging analysis. WH was the principal investigator of the study and was responsible for the study conception and interpretation of data and had final responsibility for the decision to submit for publication. All authors provided final approval for the version of the manuscript submitted for publication and agree to be accountable for the work.

## Conflict of interest

The authors declare that the research was conducted in the absence of any commercial or financial relationships that could be construed as a potential conflict of interest.

## Publisher's note

All claims expressed in this article are solely those of the authors and do not necessarily represent those of their affiliated organizations, or those of the publisher, the editors and the reviewers. Any product that may be evaluated in this article, or claim that may be made by its manufacturer, is not guaranteed or endorsed by the publisher.
