# Supplementary material for: Association of enlarged perivascular spaces with upper extremities and gait impairment: An observational, prospective cohort study
Source: Front Neurol. 2022 Oct 26;13:993979. doi: 10.3389/fneur.2022.993979 (PMC9644133; doi:10.3389/fneur.2022.993979)
Supplement: Supplementary file 1 [file Table_1.doc]

Supplement table 1 Laboratory tests in participants with different severity of EPVS

| Variables | All  (n=206) | BG-EPVS | | | CSO-EPVS | | |
| --- | --- | --- | --- | --- | --- | --- | --- |
|  |  | Low-grade  (n=145) | high-grade  (n=61) | *P* | Low-grade  (n=120) | High-grade  (n=86) | *P* |
| WBC, *10^9^/L | 6.4(5.5,7.6) | 6.3(5.5,7.5) | 6.6(5.4,8.0) | 0.267 | 6.4(5.6,7.3) | 6.5(5.4,8.2) | 0.494 |
| Neutrophil, % | 60.3±10.6 | 59.1±8.8 | 63.0±13.5 | **0.021** | 60.5±11.9 | 60.0±8.5 | 0.927 |
| Hemoglobin, g/L | 142.0(133.0,151.0) | 142.0(132.0,150.0) | 145.0(135.0,154.0) | **0.025** | 142.0(131.0,151.0) | 143.0(135.0,151.0) | 0.238 |
| Platelet count, *10^9^/L | 208.0(175.0,249.0) | 211.5(177.0,256.0) | 194.0(158.5,233.0) | 0.037 | 213.0(181.0,250.0) | 203.0(166.0,247.0) | 0.139 |
| Cholesterol, mg/dL | 172.4(145.7,201.8) | 175.1(147.1,204.7) | 166.6(135.1,194.7) | 0.102 | 171.4(144.4,203.2) | 173.8(150.2,200.1) | 0.920 |
| LDL, mg/dL | 98.2±33.8 | 98.8±34.9 | 96.7±30.7 | 0.708 | 97.6±33.0 | 99.0±35.0 | 0.501 |
| HDL, mg/dL | 38.7(34.8,46.8) | 38.9(34.8,49.1) | 38.7(34.8,44.9) | 0.160 | 38.7(34.8,47.6) | 38.7(34.8,46.4) | 0.501 |
| Triglyceride, mg/dL | 132.0(102.3,189.2) | 132.9(102.8,194.0) | 131.1(99.2,158.6) | 0.336 | 125.8(98.3,191.4) | 136.4(108.1,184.3) | 0.664 |
| AST, U/L | 20.0(17.0,25.0) | 21.0(17.0,25.0) | 19.0(17.0,24.0) | 0.245 | 21.0(18.0,25.0) | 19.0(17.0,25.0) | 0.346 |
| ALT, U/L | 20.0(15.0,26.5) | 19.0(15.0,27.0) | 20.0(15.0,27.0) | 0.837 | 20.0(16.0,28.0) | 18.0(15.0,25.0) | 0.271 |
| ALP, U/L | 71.0(60.0,84.0) | 73.0(63.0,85.0) | 67.0(58.0,79.0) | 0.039 | 70.0(60.0,87.0) | 71.0(60.0,82.0) | 0.851 |
| Fasting glucose, mmol/L | 5.6(4.8,7.0) | 5.6(4.8,7.1) | 5.9(5.0,6.9) | 0.409 | 5.8(5.0,7.1) | 5.3(4.7,6.8) | 0.102 |
| HbA1C, % | 6.0(5.6,7.1) | 5.9(5.6,7.0) | 6.2(5.8,7.1) | 0.107 | 6.0(5.6,7.4) | 5.9(5.6,6.7) | 0.386 |
| Urea, mmol/L | 5.0(4.1,5.8) | 5.0(4.1,5.7) | 4.9(4.2,5.9) | 0.714 | 5.1(4.2,5.9) | 4.9(4.1,5.6) | 0.392 |
| Creatinine, mmol/L | 63.6(55.4,73.7) | 63.5(54.7,73.6) | 65.1(56.5,73.7) | 0.583 | 63.8(55.8,73.5) | 63.5(54.8,74.3) | 0.690 |
| Uric acid, mmol/L | 328.1±98.4 | 326.1±97.2 | 332.8±101.7 | 0.995 | 338.2±104.8 | 314.2±87.3 | 0.115 |
| Homocysteine, mmol/L | 14.0(11.0,16.0) | 14.0(11.0,16.0) | 13.0(11.0,17.0) | 0.489 | 14.0(11.0,16.0) | 14.0(11.0,17.0) | 0.872 |
| PT, sec | 11.2(10.7,11.7) | 11.2(10.8,11.7) | 11.1(10.7,11.8) | 0.787 | 11.2(10.7,11.7) | 11.2(10.8,11.7) | 0.819 |
| Fibrinogen, mg/dl | 243.0(207.9,284.8) | 243.0(206.7,282.7) | 242.9(210.6,291.8) | 0.949 | 244.3(204.4,282.7) | 242.9(210.6,287.2) | 0.852 |
| INR | 0.9(0.9,1.0) | 0.9(0.9,0.9) | 0.9(0.9,0.9) | 0.753 | 0.9(0.9,0.9) | 0.9(0.9,1.0) | 0.676 |
| D-dimer, mg/L | 0.2(0.1,0.4) | 0.2(0.1,0.4) | 0.3(0.2,0.6) | 0.125 | 0.2(0.2,0.4) | 0.2(0.1,0.4) | 0.394 |

Data represent number (percentage), mean ± standard deviation, or median (interquartile range).

WBC, white blood cell; LDL, low-density lipoprotein cholesterol; HDL, high-density lipoprotein cholesterol; AST, aspartate aminotransferase; ALT, alanine transaminase; ALP, alkaline phosphatase; HbA1C, hemoglobin A1C; PT, prothrombin time; INR, international normalized ratio.
